# Effects of maternal iodine nutritional status on neurodevelopmental and cognitive function of rat offspring

**DOI:** 10.3389/fnut.2022.996092

**Published:** 2022-10-19

**Authors:** Min Fu, Wen Wu, Wenxing Guo, Qi Jin, Qi Meng, Yuanpeng Gao, Rui Yang, Ying Yang, Zihao Wang, Wanqi Zhang

**Affiliations:** ^1^Department of Nutrition and Food Science, School of Public Health, Tianjin Medical University, Tianjin, China; ^2^Tianjin Key Laboratory of Environment, Nutrition and Public Health, Center for International Collaborative Research on Environment, Nutrition and Public Health, Tianjin Medical University, Tianjin, China; ^3^Department of Endocrinology and Metabolism, Tianjin Medical University General Hospital, Tianjin, China

**Keywords:** maternal iodine excess, maternal iodine deficiency, offspring neurodevelopment, placenta, mammary gland

## Abstract

**Objectives:**

This study aimed to explore the effect of maternal iodine status on the brain development of offspring in rats. Since in human studies, the interference of environmental factors and other nutrients cannot be removed.

**Materials and methods:**

A total of 48 female Wistar rats were randomly divided into four groups: low iodine (LI), normal iodine (NI), 10-fold high iodine (10HI), and 50-fold high iodine (50HI). The rats were killed on the 15th day of pregnancy and lactation after collecting 24-h urine. The iodine concentration in 24-h urine, blood, and placenta of pregnant rats, and 24-h urine, milk, blood, and mammary glands of lactating rats was determined by inductively coupled plasma mass spectrometry. The thyroid hormone of pregnant and lactating rats was detected by chemiluminescence. The offspring were subjected to the Morris water maze on the 10th day after birth. Serum was collected to detect the thyroid hormone of offspring. The protein expression of neuroendocrine-specific protein (NSP)-A and brain-derived neurotrophic factor (BDNF) in the offspring brain were studied.

**Results:**

Iodine storage in the placenta during pregnancy and mammary glands during lactation was positively correlated with iodine intake, and iodine storage in the placenta and mammary glands in the 50HI group was significantly higher than that in the NI group (*P* = 0.045 and *P* = 0.040). Compared with the NI group, the offspring thyroid-stimulating hormone (TSH) level was significantly higher in the 10HI group (*P* = 0.046), and the FT4 level was significantly lower in the 50HI group (*P* = 0.032). The Morris water maze showed that LI and 50HI groups required longer time and distance to find the platform than the NI group (*P* < 0.001). The platform crossing numbers in the LI and 50HI groups decreased significantly (*P* < 0.001). The expression of NSP-A in offspring brain was lower in the 10HI and 50HI groups than in the NI group (*P* = 0.026 and *P* = 0,008). BDNF expression levels were significantly lower in the LI, 10HI, and 50HI groups than in the NI group (*P* < 0.001).

**Conclusion:**

Maternal iodine intake affects iodine storage in the placenta and lactating mammary gland, which in turn affects thyroid function and BDNF and NSP-A expression in the offspring.

## Introduction

Infants are more sensitive to abnormal changes in iodine concentration than other age-groups as they have the highest iodine requirements per kilogram of body weight, but the thyroid stores a small amount of iodine with only about 300 μg even in iodine-rich areas (1). Inappropriate iodine nutrition leads to thyroid dysfunction. Thyroid hormone is particularly critical for fetal and infant neurodevelopment (2, 3). However, the effect of maternal iodine nutrition status in the first 1,000 days of life on the nerve and intelligence of offspring is controversial. A 9-year follow-up of the gestational iodine cohort revealed that even mild iodine deficiency during pregnancy could have long-term adverse impacts on fetal neurocognition, which were not ameliorated by iodine sufficiency during childhood (4). A U.K. study found that when the maternal urinary iodine-to-creatinine ratio was 50–150 μg/g (which indicates mild to moderate iodine deficiency), offspring had lower scores in the verbal intelligence quotient, reading accuracy, and reading comprehension (5). However, a randomized, double-blind, and placebo-controlled trial including 832 pregnant women with mild iodine deficiency (with a median urinary iodine concentration of 131 μg/L) has found that mild iodine deficiency in pregnancy has no effect on the development of offspring. This may be because pregnant women are able to physiologically adapt to mildly low iodine intakes during pregnancy, draw from intrathyroidal iodine stores, and maintain fetal euthyroidism, allowing for normal *in utero* development (6). In addition, iodine excess during pregnancy may lead to hypothyroidism in the fetus. Rapid normalization of fetal/newborn thyroid function is necessary to prevent neurological damage (7). An experiment of rats indicated that the excess iodine leads to the impairment of learning and memory, and it may be mediated *via* the mitochondrial apoptotic pathway. Long-term repetitive excess iodine exposure affects monoamine neurotransmitters in the hippocampus of rat offspring (8). But another study has shown that neurodevelopmental and cognitive deficits in pups were mild and temporary when maternal rats were given three times the normal amount of iodine (9). Therefore, the effects of iodine deficiency and excess on nerves and growth in infants need further study.

Maternal iodine is the only source of iodine for offspring during pregnancy and exclusive breastfeeding. During pregnancy, maternal iodine is passed through the placenta to the fetus (10). After parturition, breast milk is the only source of iodine for newborns (11, 12). Therefore, detecting the iodine content in the placenta during pregnancy and mammary gland during lactation more accurately reflects the iodine intake of offspring. During critical periods of brain development (from the beginning of pregnancy to early birth), the development of the nervous system is dependent on thyroid hormones (13). Tetraiodothyronine (T4) and triiodothyronine (T3) are the main hormones produced by the thyroid and are essential for growth, development, and metabolism in vertebrates (14). Maternal hypothyroidism affects the expression of fetal and newborn *brain-derived neurotrophic factor (BDNF)* (15, 16) and *neuroendocrine-specific protein (NSP)-A* (17), both of which are important mediators of thyroid hormone and have essential roles in brain development.

In this study, different concentrations of potassium iodide (KI) were added to the drinking water of female rats to construct pregnant and lactation rats with different iodine nutritional statuses. The diets and environments of female rats in each group were consistent. The Morris water maze (MWM) test was carried out for the offspring, and the expression of *BDNF* and *NSP-A* in the brain was detected to investigate the effect of maternal iodine status on the brain development of offspring.

## Materials and methods

### Animals and treatments

In total, 48 female and 24 male Wister rats aged 4 weeks after weaning were purchased from SBF Beijing Biotechnology Co., Ltd (Beijing, China). This study was approved by the Animal Research Committee of Tianjin Hospital of ITCWM Nankai Hospital (NKYY-DWLL-2021-048). The rats were maintained in standard cages at 22 ± 2°C, with a relative humidity of 40–80% under a 12-h light/12-h dark cycle and given free access to food and water.

The female rats were randomly divided into four groups (*n* = 12 rat/group): low iodine (LI, 1 μg/d), normal iodine (NI, 6 μg/d), 10-fold high iodine (10HI, 60 μg/d), and 50-fold high iodine (50HI, 300 μg/d). The rats in the LI group were provided with deionized water daily, and the other groups were given deionized water supplemented with different concentrations of KI. All rats were given a low iodine diet with plenty of other nutrients, and the average dietary iodine content was 50 mg/kg (Trophic Animal Free High-tech Co. Ltd., Jiangsu, China, TP016ID103). The male rats were fed the same as female rats in the NI group.

After 10 weeks of conditional intervention, the female rats cohabited with males (female:male = 1:1). A total of six maternal rats were selected randomly from each group to collect 24-hour (24-h) urine at the 15th day of pregnancy, whereafter, they were anesthetized with pentobarbital (40 mg/kg, intraperitoneally), and the blood and placenta were removed and weighed. To rule out other factors affecting the growth and development of the offspring and ensure consistent breastfeeding, each maternal rat kept only five pups after delivery. The day of birth was defined as postnatal day (PND) 0. On PND10, the Morris water maze (MWM) test lasted 6 days and was performed for pups to evaluate the ability of spatial learning and memory. Rat milk and 24-h urine were collected for 2 consecutive days begining on PND15 to determine iodine levels. Then, the rats were anesthetized, and the blood and mammary glands of the maternal rats were removed and weighed, as well as the blood and brain of offspring.

### Urine

At 15 days of gestation and 15 days of lactation, six female rats in each group were placed in metabolism cages for 2 days to collect 24-h urine from the maternal rats, and then the urine weight was recorded.

### Rat milk

The maternal rats were anesthetized with isoflurane and kept anesthetized during milk extraction. Then, the rats were intramuscularly injected with 0.5 mL of veterinary prolactin. After 5 min, the nipples were rubbed gently, and about 0.5 mL of milk was collected using Pasteur straws. Milk was randomly collected three times in 2 days to determine the iodine concentration in milk.

### Measurement of iodine in tissue, urine, and serum

The iodine concentration of urine, milk, serum, placenta, and mammary glands was detected and analyzed by inductively coupled plasma mass spectrometry, ICP-MS (iCAP Q, Thermo Fisher Scientific, Frankfurt am Main, Germany) using Te for mass bias correction.

### Thyroid function tests

Thyroid-stimulating hormone (TSH), free triiodothyronine (FT3), and free tetraiodothyronine (FT4) were measured using an automatic Immulite analyzer with a chemiluminescent kit (Sophonix, Beijing, China). Before testing the sample, each kit was controlled with a calibration solution and quality control product. Then 100 μL of calibration solution and quality control product were to each reagent. The samples were tested only after successful calibration and quality control, and each item requires 100 μL serum.

### Morris water maze test

The MWM test was used to assess the ability of spatial learning and memory in pups about 10 days after birth. The MWM test was performed with the DMS-2 Morris water maze test system (Institute of Materia Medica at Chinese Academy of Medical Sciences, Beijing, China). The MWM test has two phases, namely, an acquisition test and a spatial probe trial. During the 5 consecutive days of the acquisition test, each pup was released from three random locations (except for the quadrant where the platform is). The platform was 2 cm underwater. The experiment was terminated when the pup reached the hiding platform. If the pup failed to locate the platform within the 60 s, it was gently guided onto the platform and allowed to stay there for 30 s, and the escape latency was recorded as 60 s. The spatial probe trial was tested on the 6th day of the MWM test, and the platform was removed. The test time was 60 s, and the crossing numbers on the location of the removed platform were recorded.

### HE staining

HE staining of the brain sections was performed with an HE Staining Kit (Beyotime, Nantong, China) according to the manufacturer’s instructions. Images (at × 20 magnification) were captured under an Olympus IX81 microscope (Olympus, Tokyo, Japan) in the bright-field mode.

### Nissl staining

According to the manufacturer’s instructions, the brain sections were stained with cresyl violet (Beyotime, Nantong, China). Neurons with discernable and rich Nissl staining were counted as viable neurons, whereas neurons with lost Nissl bodies and condensed cytoplasm were considered damaged neurons. Images from the cerebral cortex and hippocampal CA1 region (at × 200 magnification) were captured by an Olympus IX81 microscope (Olympus, Tokyo, Japan) in the bright-field mode, and the number of surviving neurons was analyzed by NIH ImageJ 1.61 software (National Institutes of Health, Bethesda, MD, USA).

### Western blot analysis

Brains of offspring were homogenized in RIPA buffer (20 mM Tris–HCl pH 7.5, 150 mM NaCl, 1 mM EDTA, 1% Triton-X100, 0.5% sodium deoxycholate, 1 mM PMSF, and 10 μg/ml leupeptin), incubated on ice for 15 min, and centrifuged at 14,000 g for 10 min at 4°C. Protein concentrations were determined by a Bicinchoninic Acid (BCA) Protein Assay Kit (Beyotime, Nantong, China). The protein samples were fractionated through SDS-PAGE and transferred onto PVDF membranes (Millipore, Billerica, MA, USA). The PVDF membranes were blocked with 5% BSA (Sigma, Darmstadt, Germany) in 1 × Tris-buffered saline Tween for 1 h at room temperature. The membranes were incubated with primary antibodies of mouse anti-NSP-A (1:500; Santa Cruz, CA, USA), rabbit anti-BDNF (1:1000; Bioss, Beijing, China), and rabbit anti-GAPDH (loading control, 1:5000; Bioss, Beijing, China) overnight at 4°C. After washing with TBST, they were incubated with relational horseradish peroxidase [HRP]-conjugated secondary antibodies for 1 h at room temperature. Then, the proteins were detected by chemiluminescence reagents (Sparkjade, Shandong, China) and observed using a ChemiDocTM XRS + Imaging System (Bio-Rad, Hercules, CA, USA). The protein levels were quantified by densitometry using NIH ImageJ 1.61 software (National Institutes of Health, Bethesda, MD, USA).

### Immunohistochemistry

After the brains of the offspring were removed, they were immediately fixed with 4% paraformaldehyde and then embedded in paraffin. The sections were dewaxed and rehydrated, and the next steps were performed with rabbit-specific HRP/DAB (ABC) IHC Detection Kit ab64261(Abcam, Cambridgeshire, UK). The sections of brains were incubated with a primary antibody of rabbit mouse anti-NSP-A (1:100; Santa Cruz, CA, USA) and rabbit anti-BDNF (1:100; Bioss, Beijing, China) overnight at 4°C. Then, the biotinylated secondary antibody was bound to the primary antibody, and the HRP-labeled streptavidin was bound to the secondary antibody. The HRP produced a brown-colored substance at the site of primary antibody binding by reacting with DAB. The images were obtained with an inverted microscope (IX81; Olympus, Tokyo, Japan).

### Statistical analysis

The statistical software package SPSS Statistics version 20.0 (Armonk, NY, USA) and GraphPad Prism v7 (GraphPad Software Inc., San Diego, CA, USA) were used for statistical analysis. The variables conforming to a normal distribution were expressed as means ± SD. Comparisons between different groups were performed by a one-way analysis of variance (ANOVA), followed by a Student–Newman–Keuls test for multiple comparisons. The variables without normal distributions were expressed as M (P25, P75), and comparisons between groups were performed by using a Kruskal–Wallis test. Repeated-measures data of escape latency in the MWM test were compared by ANOVA. *P* < 0.05 was considered statistically significant.

## Result

### Iodine nutritional status and thyroid function during pregnancy in rats

Serum was collected on the 15th day of pregnancy to detect serum iodine, and 24-h urine was collected from the metabolic cage for 2 days. The serum total iodine concentration (STIC) and 24-h urinary iodine concentration (UIC) of the 50HI group were significantly higher than those of the NI group (*P* = 0.020 and *P* = 0.020, Table 1).

**TABLE 1:** Serum iodine and 24-h urinary iodine concentration of pregnant rats.

Group	N	STIC (μg/l)	24-h UIC (μg/l)
LI	6	15.85 (10.31, 20.02)	87.45 (78.71, 95.64)
NI	6	42.28 (37.09, 49.17)	369.96 (312.10, 433.28)
10HI	6	144.99 (129.17, 164.23)	3569.18 (3287.96, 3793.56)
50HI	6	757.23 (703.17, 910.12)^a^	12286.04 (11470.80, 13362.59)^b^
*P*-value		<0.001	<0.001

STIC, serum total iodine concentration; UIC, urinary iodine concentration.

^a^Compared with the NI group, *P* = 0.020.

^b^Compared with the NI group, *P* = 0.020.

Results of the thyroid function test during pregnancy are shown in Table 2. Compared with the NI group, serum FT4 levels were significantly increased in the 10HI and 50HI groups (*P* = 0.010 and *P* = 0.012).

**TABLE 2:** TSH, FT3, and FT4 concentration of pregnant rats.

Group	N	TSH (mIU/L)	FT3 (pg/mL)	FT4 (pg/mL)
LI	6	0.02 ± 0.01	2.01 ± 0.28	6.00 ± 0.92
NI	6	0.02 ± 0.02	1.81 ± 0.13	7.11 ± 1.38
10HI	6	0.01 ± 0.01	1.85 ± 0.31	11.96 ± 2.95^a^
50HI	6	0.04 ± 0.04	2.02 ± 0.32	11.49 ± 4.30^b^
*P*-value		0.734	0.400	0.008

TSH, thyroid-stimulating hormone; FT3, free triiodothyronine; FT4, free tetraiodothyronine.

^a^Compared with the NI group, *P* = 0.010.

^b^Compared with the NI group, *P* = 0.012.

### Iodine stored in the placenta during pregnancy in rats

The placenta iodine concentration was positively correlated with iodine intake, and there was a significant change between the NI and 50HI groups (*P* = 0.012). With the increase in iodine intake, the placental iodine storage tended to increase, and the placental iodine storage in the 50HI group was significantly higher than that in the NI group (*P* = 0.045, Table 3).

**TABLE 3:** Concentration and content of iodine in the placenta of pregnant rats.

Group	n	Placenta	
		
		Concentration (μg/g)	Storage (μg)
LI	6	0.02 (0.01, 0.08)	0.16 (0.04, 0.47)
NI	6	0.07 (0.04, 0.11)	0.59 (0.26, 0.87)
10HI	6	0.17 (0.11, 0.24)	0.32 (0.16, 0.90)
50HI	6	0.60 (0.37, 0.78)^a^	3.14 (0.65, 4.98)^b^
*P*-value		<0.001	0.022

^a^Compared with the NI group, *P* = 0.012.

^b^Compared with the NI group, *P* = 0.045.

### Iodine nutritional status and thyroid function during lactation in rats

On the 15th day of lactation, we collected 24-h urine, serum, and milk from the maternal rats. As shown in Table 4, maternal 24h-UIC, STIC, and breast milk iodine concentration (BMIC) increased as iodine intake increased. The STIC, UIC, and BMIC of the 50HI groups were significantly higher than those of the NI group (*P* = 0.020, *P* = 0.017 and *P* = 0.017).

**TABLE 4:** Iodine concentrations in 24-h urine, blood, and milk of maternal rats.

Group	N	STIC (μg/l)	24-h UIC^a^ (μg/l)	BMIC^b^ (μg/l)
LI	6	11.98 (79.51, 90.58)	85.56 (79.51, 90.58)	58.30 (48.26, 91.36)
NI	6	31.96 (28.61, 39.97)	206.61 (189.19, 298.43)	132.27 (115.34, 178.72)
10HI	6	210.09 (189.34, 235.65)	4143.90 (3333.18, 4880.84)	1057.15 (870.07, 1277.13)
50HI	6	654.93 (586.16, 684.29)^c^	13202.03 (12141.22, 15248.98)^d^	9801.73 (9296.99, 12279.88)^e^
*P*-value		<0.001	<0.001	<0.001

UIC, urinary iodine concentration; STIC, serum total iodine concentration; BMIC, breast milk iodine concentration.

^a^Urinary iodine concentration was average value over 2 days.

^b^Breast milk iodine concentration was the average value of three random samples.

^c^Compared with the NI group, *P* = 0.020.

^d^Compared with the NI group, *P* = 0.017.

^e^Compared with the NI group, *P* = 0.017.

The thyroid function of the rats during lactation is shown in Table 5. Compared with the NI group, serum FT4 levels were significantly increased when exposed to 10HI and 50HI doses (*P* = 0.037 and *P* < 0.001).

**TABLE 5:** TSH, FT3, and FT4 concentration of lactating rats.

Group	N	TSH (mIU/L)	FT3 (pg/mL)	FT4 (pg/mL)
LI	6	0.01 ± 0.01	1.72 ± 0.45	8.45 ± 2.58
NI	6	0.05 ± 0.15	1.68 ± 0.45	9.76 ± 2.01
10HI	6	0.03 ± 0.05	1.60 ± 0.21	13.26 ± 3.78^a^
50HI	6	0.03 ± 0.02	1.98 ± 0.25	18.84 ± 5.26^b^
*P*-value		0.801	0.313	<0.001

TSH, thyroid-stimulating hormone; FT3, free triiodothyronine; FT4, free tetraiodothyronine.

^a^Compared with the NI group, *P* = 0.037.

^b^Compared with the NI group, *P* < 0.001.

### Iodine stored in mammary glands during lactation in rats

During lactation, the main iodinated tissue is the mammary gland, in addition to the thyroid (18). The amount of iodine stored in the mammary gland more directly reflects the iodine intake of the newborn. Results of iodine concentration and storage in the mammary gland are shown in Table 6. With the increase in maternal iodine intake, the iodine concentration and storage in the mammary gland also increased, and the difference of the concentration (*P* = 0.017) and storage (*P* = 0.040) of iodine in the mammary gland between the 50HI group and the NI group was significant.

**TABLE 6:** Iodine concentration and content in the mammary gland of lactating rats.

Group	N	Mammary gland	
		
		Concentration (μg/g)	Storage (μg)
LI	6	0.04 (0.02, 0.07)	0.75 (0.30, 1.16)
NI	6	0.06 (0.05, 0.10)	1.17 (0.66, 2.82)
10HI	6	0.48 (0.30, 0.80)	6.55 (2.86, 9.47)
50HI	6	5.22 (0.76, 8.50)^a^	90.37 (9.08, 158.53)^b^
*P*-value		<0.001	0.003

^a^Compared with the NI group, *P* = 0.017.

^b^Compared with the NI group, *P* = 0.040.

### Iodine nutritional status and thyroid function of rat offspring

Our results show that the iodine nutrition status of the mother affected the iodine nutrition status and thyroid function of the offspring. One pup per female was randomly selected for brachial artery blood sampling for serum iodine and thyroid function (Table 7). As iodine intake increased, the STIC gradually increased, and the difference in the STIC between the 50HI group and the NI group was significant (*P* = 0.003). Compared with the NI group, the TSH level was significantly higher in the 10HI group (*P* = 0.046), and the FT4 level was significantly lower in the 50HI group (*P* = 0.032).

**TABLE 7:** Evaluation of iodine status and thyroid function of rat offspring.

Group	N	Iodine nutrition	Thyroid function		
			
		STIC (μg/L)	TSH (mIU/L)	FT3 (pg/mL)	FT4 (pg/mL)
LI	6	64.41 (51.66, 122.30)	0.005 ± 0.002	2.36 ± 0.48	23.41 ± 4.23
NI	6	105.79 (73.18, 126.78)	0.006 ± 0.001	1.91 ± 0.45	24.65 ± 9.51
10HI	6	635.36 (481.11, 665.84)	0.008 ± 0.03^b^	1.74 ± 0.44	21.77 ± 6.91
50HI	6	2956.58 (2130.11, 4486.79)^a^	0.007 ± 0.002	1.82 ± 0.43	15.70 ± 8.78^c^
*P*-value		<0.001	0.063	0.075	0.154

STIC, serum total iodine concentration; TSH, thyroid-stimulating hormone; FT3, free triiodothyronine; FT4, free tetraiodothyronine.

^a^Compared with the NI group, *P* = 0.003.

^b^Compared with the NI group, *P* = 0.046.

^c^Compared with the NI group, *P* = 0.032.

### Effects of maternal iodine malnutrition on the brain development of rat offspring

**FIGURE 1:**
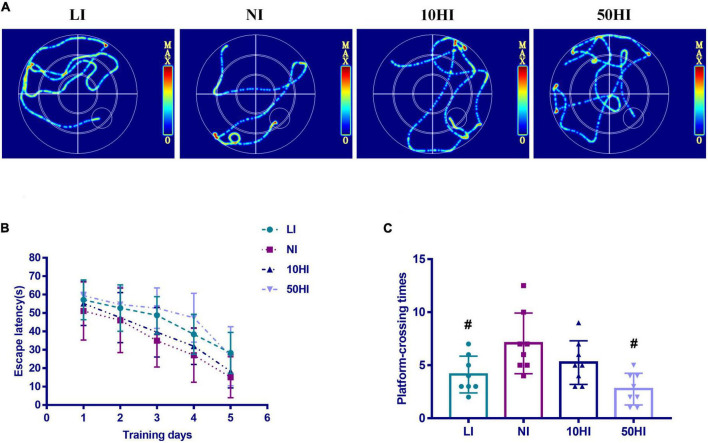
**(A)** Representative swimming paths. **(B)** Escape latency during the spatial acquisition phase of the MWM test. **(C)** Crossing numbers on the location of the removed platform during the spatial probe phase of the MWM test. (Values are shown as mean ± SD. *N* = 30 per group. Repeated-measures data of escape latency in the MWM test were compared using the repeated-measures ANOVA. One-way ANOVA and LSD tests were used to compare the number of crossing platforms between groups. ^#^*P* < 0.05 compared with the NI group).

The MWM test was performed to evaluate spatial learning and memory ability. Representative swimming paths of rat offspring in the LI, NI, 10HI, 50HI groups on the 5th day (Figure 1A). Decreased values for escape latency and increased values for the platform crossing numbers, respectively, indicate better spatial learning ability and better memory ability. The MWM test was performed on five pups per female. With the increase in training time, escape latency decreased in all four groups (Figure 1B). Repeated-measures ANOVA showed that the LI and 50HI groups required longer time and distance to find the platform than the NI group (*P* < 0.05). The poor memory skill of LI and 50HI groups was also shown by the fewer platform crossing numbers measured on the 6th day (Figure 1C). The results of the MWM test showed that maternal iodine deficiency and excess affect the brain development of offspring.

### Effects of maternal iodine malnutrition on neuronal morphology in rat offspring

**FIGURE 2:**
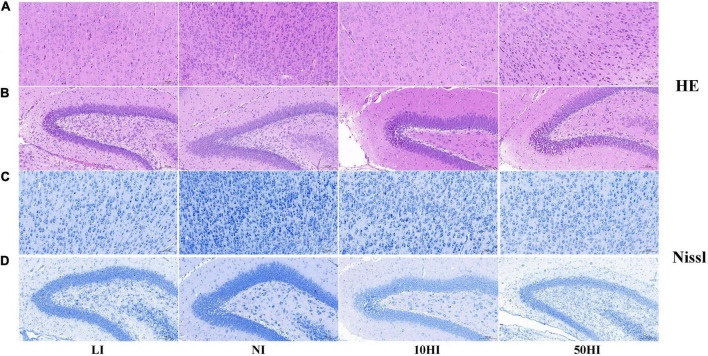
Representative micrographs of HE staining in the pup cerebral cortex **(A)** and hippocampal CA1 region **(B)**. Representative micrographs of Nissl staining in the pup cerebral cortex **(C)** and hippocampal CA1 region **(D)**. Scale bars: 50 mm.

HE staining was performed to observe neuronal morphology in the cerebral cortex and hippocampal CA1 region. In the NI group, the majority of the neurons contained abundant cytoplasm with well-defined nuclei. Some of the neurons had disordered tissue arrangement in the LI, 10HI, and 50HI groups. Irregular cell morphology, pyknosis, unclear nucleolus, and deep staining of cytoplasm were found (Figures 2A,B). Nissl staining was performed to quantify neuronal survival. The neurons in the cerebral cortex and hippocampal CA1 region of the four groups were arranged densely and orderly, with abundant Nissl bodies in the cytoplasm. There is also no significant difference among the four groups (Figures 2C,D).

### Effects of maternal iodine malnutrition on the expression of BDNF and NSP-A in the brain of the offspring

**FIGURE 3:**
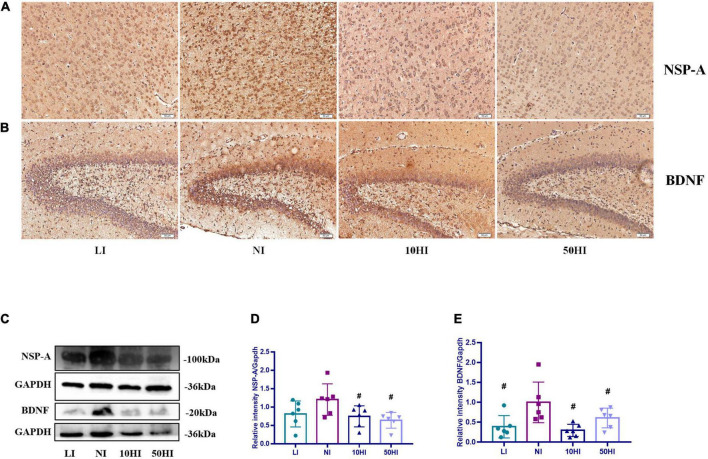
**(A)** Localization of NSP-A in the cerebral cortex. **(B)** Localization of BDNF in the hippocampal DG region. Scale bars: 50 μm. **(C)** Representative Western blot results of NSP-A and BDNF. Bar graphs show the semiquantitative levels of NSP-A **(D)** and BDNF **(E)** determined by band density analysis. (Values are shown as mean ± SD. *N* = 6 per group. Comparisons among different groups were performed by one-way ANOVA, followed by an LSD test for multiple comparisons. ^#^*P* < 0.05 compared with the NI group).

NSP-A and BDNF are affected by maternal thyroid hormones and have essential roles in brain development. Photomicrographs of the immunohistochemistry-stained brains of offspring on PND15 showed positive expression of NSP-A in the cerebral cortex (Figure 3A) and BDNF in the hippocampal CA1 region (Figure 3B) in all treatment groups. The general protein expressions of NSP-A and BDNF are shown in Figure 3C. The expression of NSP-A was lower in the 10HI and 50HI groups than in the NI group (*P* < 0.05, Figure 3D). The BDNF expression level was significantly lower in the LI, 10HI, and 50HI groups than in the NI group (*P* < 0.05, Figure 3E).

## Discussion

In this study, we examined placenta iodine storage during pregnancy and mammary gland iodine storage during lactation in female rats, which more directly reflect iodine intake levels in the early life of offspring. Different concentrations of KI were added to the drinking water of female rats, and the diets and environments of maternal rats in each group were consistent. Our study clarifies the effects of maternal iodine nutrition levels during pregnancy and lactation on offspring brain development, overcoming the inability to control the effects of environmental and other nutrients on offspring brain development in human studies in addition to iodine.

Our findings showed that maternal iodine intake impacted the iodine content in the placenta. The placental iodine storage in the 50HI group was significantly higher than that in the NI group. Previous studies also support that the placenta stores iodine in a concentration-dependent manner and serves as long-term storage and supply (19–21). There was no statistically significant difference between the LI and 10HI groups and the NI group because the placenta is an important organ that forms a barrier where the maternal and fetal exchange occurs, which has a role in regulating abnormal maternal iodine status (22). Therefore, testing only maternal iodine intake and UIC in human studies does not accurately reflect the iodine received by the fetus.

Breast milk is currently considered the ideal natural food for the growth and development of infants. The energy and nutrients in breast milk meet the growth and development needs of infants up to 6 months of age (23). The calcium content in milk remains stable, regardless of whether the lactating mother has sufficient dietary calcium or not. When dietary calcium intake is insufficient, in order to maintain the milk calcium content constant, it is necessary to use the maternal bone calcium (24, 25). This explains mammary glands have certain regulation ability to the abnormal nutrients of the maternal body. During lactation, there is increased expression of NIS, which mediates iodine uptake into the mammary gland, and a portion of the body iodine is transferred to the mammary gland (26). It is uncertain whether breast NIS compensates for milk iodine. However, the results of this our study showed a positive correlation between the BMIC and iodine intake, but only the 50HI group was significantly higher than the NI group (*P* = 0.017). Therefore, the compensatory ability of the mammary gland for iodine deficiency and excess during lactation needs further study.

Maternal thyroid hormone plays an important role in fetal brain development. It regulates brain morphological and biochemical changes before the fetal thyroid is functioning, which occurs in the first trimester of pregnancy (27, 28). During pregnancy and early lactation, iodine of the offspring is derived completely from the mother. Excessive and insufficient iodine may cause hypothyroidism (29, 30). In the fetus and the infant, iodine deficiency affects brain development, leading to a decline in mental activity and, in extreme cases, cretinism (31–33). Studies on the effects of iodine deficiency and excess on infant nerve and growth are scarce. In human studies, it is impossible to avoid the interference of nutrients other than iodine and the environment in the experimental results. For example, the energy required for neurodevelopment is maintained by glucose, iron, copper, zinc, and selenium. The structure and composition of the brain are influenced by protein, long-chain polyunsaturated fatty acid deposits, and folate *via* neurulation. The process of neural cell differentiation is supported by micronutrients, such as iodine and zinc (34). Perchlorate is an environmental contaminant that interferes with iodine uptake into the thyroid to affect brain development (35). The animal experiment we carried out fully screened for the effects of these confounding factors.

BNDF and NSP-A could be used to assess brain development of offspring at the molecular level. BDNF is a neurotrophic protein that plays a critical role in brain development and is involved in neurogenesis, neuronal differentiation, synaptogenesis, and memory formation and consolidation (36–39). NSP-A is believed to be an important mediator of thyroid hormone action during brain development and is involved in neuronal differentiation and axon guidance (40). A previous study has shown that on PND7, when iodine deficiency (iodine intake was 1.5 mg/d) and mild iodine overdose (iodine intake was 15–16 mg/d) occurred, BNDF protein expression was significantly reduced, while NSP-A protein expression was significantly increased. But on PND45, no significant increase in the NSP-A level was observed when iodine intake was 15–16 mg/d (9). On this basis, our study added to explore the situation of severe iodine excess for offspring on PND15. When iodine status was abnormal, the protein expression of BNDF was significantly lower than that of the NI group. However, our results showed that the expression of NSP-A in the 10HI and 50HI groups was lower than that in the NI group. Dowling et al. (17) showed that the expression of NSP-A was regulated by thyroid hormone, and the expression of NSP-A was positively correlated with T4 in the fetal rat brain cortex. According to the thyroid function of the maternal rats, as given in Tables 2, 5, FT4 was significantly reduced in the 10 and 50HI groups. Therefore, when iodine is excessive, reduced FT4 may lead to reduced expression of NSP-A. Since there was no follow-up study of the offspring, we could not determine whether the brain damage in the offspring of the LI, 10HI, and 50HI groups was also temporary. This needs to be further discussed in future studies.

## Conclusion

For maternal rats, iodine storage in the placenta during pregnancy and in the mammary gland during lactation is associated with iodine intake. However, the placenta and lactating mammary gland have compensatory regulatory effects on iodine deficiency and iodine excess. Maternal iodine nutritional status during pregnancy and lactation affects neurodevelopment and the cognitive level of offspring by affecting their thyroid function.

## Data availability statement

The raw data supporting the conclusions of this article will be made available by the authors, without undue reservation.

## Ethics statement

The animal study was reviewed and approved by the Animal Research Committee of Tianjin Hospital of ITCWM Nankai Hospital.

## Author contributions

WZ, MF, WW, and WG conceived and designed the experiments. MF, WW, WG, YG, RY, QJ, QM, YY, and ZW performed the experiments. MF, YG, and QJ analyzed the data. MF, WG, and YG wrote the manuscript. All authors contributed to the article and approved the submitted version.

## References

[1] Zimmermann MB. Are weaning infants at risk of iodine deficiency even in countries with established iodized salt programs? *Nestle Nutr Inst Workshop Ser.* (2012) 70:137–46. 10.1159/000337678 25825303

[2] Farebrother J Zimmermann MB Andersson M. Excess iodine intake: sources, assessment, and effects on thyroid function. *Ann N Y Acad Sci.* (2019) 1446:44–65. 10.1111/nyas.14041 30891786

[3] Pearce EN Lazarus JH Moreno-Reyes R Zimmermann MB. Consequences of iodine deficiency and excess in pregnant women: an overview of current knowns and unknowns. *Am J Clin Nutr.* (2016) 104 (Suppl. 3):918s–23s. 10.3945/ajcn.115.110429 27534632 PMC5004501

[4] Hynes KL Otahal P Hay I Burgess JR. Mild iodine deficiency during pregnancy is associated with reduced educational outcomes in the offspring: 9-year follow-up of the gestational iodine cohort. *J Clin Endocrinol Metab.* (2013) 98:1954–62. 10.1210/jc.2012-4249 23633204

[5] Bath SC Steer CD Golding J Emmett P Rayman MP. Effect of inadequate iodine status in UK pregnant women on cognitive outcomes in their children: results from the Avon Longitudinal Study of Parents and Children (ALSPAC). *Lancet (London, England).* (2013) 382:331–7. 10.1016/S0140-6736(13)60436-5 23706508

[6] Gowachirapant S Jaiswal N Melse-Boonstra A Galetti V Stinca S Mackenzie I Effect of iodine supplementation in pregnant women on child neurodevelopment: a randomised, double-blind, placebo-controlled trial. *Lancet Diab Endocrinol.* (2017) 5:853–63. 10.1016/S2213-8587(17)30332-7 29030199

[7] Thomas Jde V Collett-Solberg PF. Perinatal goiter with increased iodine uptake and hypothyroidism due to excess maternal iodine ingestion. *Horm Res.* (2009) 72:344–7. 10.1159/000249162 19844123

[8] Cui Y Zhang B Zhang Z Nie J Liu H. Long-term repetitive exposure to excess iodine induces mitochondrial apoptosis, and alters monoamine neurotransmitters in hippocampus of rats of different genders. *Toxicol Res.* (2021) 10:975–82. 10.1093/toxres/tfab082 34733482 PMC8557656

[9] Zhang L Teng W Liu Y Li J Mao J Fan C Effect of maternal excessive iodine intake on neurodevelopment and cognitive function in rat offspring. *BMC Neurosci.* (2012) 13:121. 10.1186/1471-2202-13-121 23043431 PMC3479063

[10] Burns R O’Herlihy C Smyth PP. The placenta as a compensatory iodine storage organ. *Thyroid.* (2011) 21:541–6. 10.1089/thy.2010.0203 21417918

[11] Velasco I Bath SC Rayman MP. Iodine as essential nutrient during the first 1000 days of life. *Nutrients.* (2018) 10:290. 10.3390/nu10030290 29494508 PMC5872708

[12] Schwarzenberg SJ Georgieff MK. Advocacy for improving nutrition in the first 1000 days to support childhood development and adult health. *Pediatrics.* (2018) 141:e20173716. 10.1542/peds.2017-3716 29358479

[13] Bougma K Aboud FE Harding KB Marquis GS. Iodine and mental development of children 5 years old and under: a systematic review and meta-analysis. *Nutrients.* (2013) 5:1384–416. 10.3390/nu5041384 23609774 PMC3705354

[14] Carvalho DP Dupuy C. Thyroid hormone biosynthesis and release. *Mol Cell Endocrinol.* (2017) 458:6–15. 10.1016/j.mce.2017.01.038 28153798

[15] Liu D Teng W Shan Z Yu X Gao Y Wang S The effect of maternal subclinical hypothyroidism during pregnancy on brain development in rat offspring. *Thyroid.* (2010) 20:909–15. 10.1089/thy.2009.0036 20615128

[16] Lasley SM Gilbert ME. Developmental thyroid hormone insufficiency reduces expression of brain-derived neurotrophic factor (BDNF) in adults but not in neonates. *Neurotoxicol Teratol.* (2011) 33:464–72. 10.1016/j.ntt.2011.04.001 21530650

[17] Dowling AL Iannacone EA Zoeller RT. Maternal hypothyroidism selectively affects the expression of neuroendocrine-specific protein A messenger ribonucleic acid in the proliferative zone of the fetal rat brain cortex. *Endocrinology.* (2001) 142:390–9. 10.1210/endo.142.1.7871 11145602

[18] Portulano C Paroder-Belenitsky M Carrasco N. The Na+/I- symporter (NIS): mechanism and medical impact. *Endocr Rev.* (2014) 35:106–49. 10.1210/er.2012-1036 24311738 PMC3895864

[19] Karaoglan M İşbilen E. The role of placental iodine storage in the neonatal thyroid stimulating hormone surge: iodine as a driving force to adapt the terrestrial life. *J Endocrinol Invest.* (2021) 44:1041–52. 10.1007/s40618-020-01399-y 32860210

[20] Neven KY Marien CBD Janssen BG Roels HA Waegeneers N Nawrot TS Variability of iodine concentrations in the human placenta. *Sci Rep.* (2020) 10:161. 10.1038/s41598-019-56775-3 31932629 PMC6957482

[21] Neven KY Cox B Vrijens K Plusquin M Roels HA Ruttens A Determinants of placental iodine concentrations in a mild-to-moderate iodine-deficient population: an ENVIRONAGE cohort study. *J Transl Med.* (2020) 18:426. 10.1186/s12967-020-02601-8 33172470 PMC7654607

[22] Peng S Li C Xie X Zhang X Wang D Lu X Divergence of iodine and thyroid hormones in the fetal and maternal parts of human-term placenta. *Biol Trace Elem Res.* (2020) 195:27–38. 10.1007/s12011-019-01834-z 31502179

[23] Fischer Fumeaux CJ Garcia-Rodenas CL De Castro CA Courtet-Compondu MC Thakkar SK Beauport L Longitudinal analysis of macronutrient composition in preterm and term human milk: a prospective cohort study. *Nutrients.* (2019) 11:1525. 10.3390/nu11071525 31277502 PMC6683284

[24] Bae YJ Kratzsch J. Vitamin D and calcium in the human breast milk. *Best Pract Res Clin Endocrinol Metab.* (2018) 32:39–45. 10.1016/j.beem.2018.01.007 29549958

[25] Bauman DE Currie WB. Partitioning of nutrients during pregnancy and lactation: a review of mechanisms involving homeostasis and homeorhesis. *J Dairy Sci.* (1980) 63:1514–29. 10.3168/jds.S0022-0302(80)83111-0 7000867

[26] Micali S Bulotta S Puppin C Territo A Navarra M Bianchi G Sodium iodide symporter (NIS) in extrathyroidal malignancies: focus on breast and urological cancer. *BMC Cancer.* (2014) 14:303. 10.1186/1471-2407-14-303 24884806 PMC4019362

[27] Morreale de Escobar G Obregon MJ Escobar del Rey F. Role of thyroid hormone during early brain development. *Eur J Endocrinol.* (2004) 151(Suppl. 3):U25–37. 10.1530/eje.0.151u025 15554884

[28] Zoeller RT Rovet J. Timing of thyroid hormone action in the developing brain: clinical observations and experimental findings. *J Neuroendocrinol.* (2004) 16:809–18. 10.1111/j.1365-2826.2004.01243.x 15500540

[29] Hwang S Lee EY Lee WK Shin DY Lee EJ. Correlation between iodine intake and thyroid function in subjects with normal thyroid function. *Biol Trace Element Res.* (2011) 143:1393–7. 10.1007/s12011-011-8997-x 21340678

[30] Orito Y Oku H Kubota S Amino N Shimogaki K Hata M Thyroid function in early pregnancy in Japanese healthy women: relation to urinary iodine excretion, emesis, and fetal and child development. *J Clin Endocrinol Metab.* (2009) 94:1683–8. 10.1210/jc.2008-2111 19258403

[31] Verheesen RH Schweitzer CM. Iodine deficiency, more than cretinism and goiter. *Med Hypotheses.* (2008) 71:645–8. 10.1016/j.mehy.2008.06.020 18703293

[32] Chen ZP Hetzel BS. Cretinism revisited. *Best Pract Res Clin Endocrinol Metab.* (2010) 24:39–50. 10.1016/j.beem.2009.08.014 20172469

[33] Zhang L Sun YN Li YM Lin LX Ye Y Yan YQ Effect of different iodine nutrition on cerebellum Pcp-2 in rat offspring during lactation. *Biol Trace Elem Res.* (2011) 143:1629–39. 10.1007/s12011-011-8991-3 21344292

[34] Lockyer F McCann S Moore SE. Breast milk micronutrients and infant neurodevelopmental outcomes: a systematic review. *Nutrients.* (2021) 13:3848. 10.3390/nu13113848 34836103 PMC8624933

[35] Gilbert ME Hassan I Wood C O’Shaughnessy KL Spring S Thomas S Gestational exposure to perchlorate in the rat: thyroid hormones in fetal thyroid gland, serum, and brain. *Toxicol Sci J Soc Toxicol.* (2022) 188:117–30. 10.1093/toxsci/kfac038 35385113 PMC10732305

[36] Wang Y Su B Xia Z. Brain-derived neurotrophic factor activates ERK5 in cortical neurons via a Rap1-MEKK2 signaling cascade. *J Biol Chem.* (2006) 281:35965–74. 10.1074/jbc.M605503200 17003042

[37] Lewin GR Barde YA. Physiology of the neurotrophins. *Annu Rev Neurosci.* (1996) 19:289–317. 10.1146/annurev.ne.19.030196.001445 8833445

[38] Lu B Figurov A. Role of neurotrophins in synapse development and plasticity. *Rev Neurosci.* (1997) 8:1–12. 10.1515/REVNEURO.1997.8.1.1 9402641

[39] Heldt SA Stanek L Chhatwal JP Ressler KJ. Hippocampus-specific deletion of BDNF in adult mice impairs spatial memory and extinction of aversive memories. *Mol Psychiatry.* (2007) 12:656–70. 10.1038/sj.mp.4001957 17264839 PMC2442923

[40] Senden NH Timmer ED Boers JE van de Velde HJ Roebroek AJ Van de Ven WJ Neuroendocrine-specific protein C (NSP-C): subcellular localization and differential expression in relation to NSP-A. *Eur J Cell Biol.* (1996) 69:197–213. 8900485

